# Perasafe, Virkon and bleach are bactericidal for *Burkholderia pseudomallei*, a select agent and the cause of melioidosis

**DOI:** 10.1016/j.jhin.2010.06.026

**Published:** 2011-02

**Authors:** V. Wuthiekanun, G. Wongsuwan, S. Pangmee, N. Teerawattanasook, N.P. Day, S.J. Peacock

**Affiliations:** a Mahidol–Oxford Tropical Medicine Research Unit, Faculty of Tropical Medicine, Mahidol University, Bangkok, Thailand; b Medical Department, Sappasithiprasong Hospital, Ubon Ratchathani, Thailand; c Department of Microbiology and Immunology, Faculty of Tropical Medicine, Mahidol University, Bangkok, Thailand

Madam,

Our diagnostic and research microbiology laboratories are within Sappasithiprasong Hospital, a 1000-bedded district hospital in northeast Thailand. Every year we process hundreds of clinical samples that contain *Burkholderia pseudomallei*. This category B select agent is a Gram-negative, non-spore-forming bacillus and the cause of melioidosis.^1,2^ Furthermore, up to 80% of soil samples in northeast Thailand contain *B. pseudomallei* at counts that can reach >10 000 colony-forming units (cfu)/g of soil.^3–5^ Hospital floors become contaminated with soil particles deposited from footwear, and some connecting corridors on the ground floor of the hospital are intermittently flooded during the rainy season. Our hospital uses reusable endotracheal and nasogastric tubes, and these will often become contaminated with *B. pseudomallei*, since 50% of patients with melioidosis develop pneumonia, the median *B. pseudomallei* count in respiratory secretions has been reported to be 1.1 × 10^5^ cfu/mL, and seriously ill patients with melioidosis rarely die in the absence of an endotracheal tube.^6^

We are evaluating the bactericidal efficacy of chemical germicides used to decontaminate our microbiology laboratories and reusable medical equipment. Here, we report the results of a pilot study on the efficacy of Perasafe™ (Antec International Ltd, UK), Virkon™ (Antec International Ltd) and bleach against *B. pseudomallei*. Perasafe, a commercial peracetic acid disinfectant, is used to decontaminate reusable endotracheal and nasogastic tubes. A Perasafe solution is made each week and aliquots used multiple times over a period of five days. Used tubes are taken to a central area for cleaning to remove organic material followed by immersion for 30 min in 0.2% Perasafe and then rinsing using prechlorinated tap water. A 1% (w/v) solution of Virkon is used in the microbiology laboratory for environmental cleaning and for initial decontamination of disposables such as swabs prior to incineration. Bleach is inexpensive, and may be used during clean up of spills involving cultured material.

Solutions of 0.2% Perasafe, 1% Virkon and 0.5% sodium hypochlorite (bleach) (Bornnet Corporation Company Ltd, Bangkok, Thailand) were prepared as recommended by the manufacturer. In addition, aliquots of Perasafe being used by the hospital to decontaminate reusable equipment were collected at the end of days 1, 3 and 5. Bactericidal activity was evaluated for 10 clinical isolates of *B. pseudomallei* cultured from patients presenting to our hospital. The efficacy of the three chemical germicides was tested using a suspension assay with a contact time of 30 min. A bacterial suspension was prepared as follows. Six colonies picked from Columbia agar (Oxoid, Basingstoke, UK) were resuspended in 10 mL of tryptone soya broth (TSB, Oxoid) and incubated overnight at 37 °C in air with shaking at 150 rpm. Each broth was then diluted 1:10 in fresh TSB and incubated as before for 4 h to achieve a turbidity of 2.0–2.1 MacFarland (∼2 × 10^9^ cfu/mL). Each bacterial suspension was diluted to 1:10 by adding 130 μL of suspension to 1170 μL of 0.2% Perasafe, 1% Virkon, 0.5% sodium hypochlorite or distilled water; each was prepared in duplicate for all isolates. Tubes were maintained at room temperature (25 °C) for 30 min and then centrifuged at 10,000 rpm for 2 min. Pellets were washed three times with sterile distilled water prior to resuspension to a volume of 1300 μL. The viable colony count was performed on Columbia agar. A 100 μL volume of neat solution from each tube was spread plated in triplicate on to Columbia agar and the remaining solution transferred into 9 mL TSB which was incubated at 37 °C in air for 48 h before subculture in triplicate on to Columbia agar. All agar plates were incubated at 37 °C in air, inspected daily, and a colony count performed on day 4. The initial bacterial inoculum was defined using colony counts as a median of 2.2 × 10^8^ cfu/mL (range: 1.4 × 10^8^–3.7 × 10^8^).

The three germicides were 100% bactericidal for all 10 isolates under these conditions, with no growth observed on any of the agar plates on day 4. This included all Perasafe samples that were in use by the hospital for cleaning of reusable equipment. There was no reduction in inoculum for the control growth tube. We conclude that the use of Perasafe to clean reusable endotracheal and nasogastric tubes appears to be effective, and that 1% Virkon with a contact time of 30 min will be cidal for *B. pseudomallei*. We stress that cleaning of reusable equipment is critical, together with the disposal of tubes that have physical signs of wear. This is an important finding for melioidosis-endemic, resource-restricted settings where reusable equipment is commonplace. We are not yet reassured that bleach is effective for environmental cleaning since the contact times used in this pilot study would not be achieved during routine surface cleaning, and studies are ongoing using shorter contact times.
